# Pseudouridine-mediated translation control of mRNA by methionine aminoacyl tRNA synthetase

**DOI:** 10.1093/nar/gkaa1178

**Published:** 2020-12-10

**Authors:** Ofri Levi, Yoav S Arava

**Affiliations:** Faculty of Biology, Technion – Israel Institute of Technology, Haifa 3200003, Israel; Faculty of Biology, Technion – Israel Institute of Technology, Haifa 3200003, Israel

## Abstract

Modification of nucleotides within an mRNA emerges as a key path for gene expression regulation. Pseudouridine is one of the most common RNA modifications; however, only a few mRNA modifiers have been identified to date, and no one mRNA pseudouridine reader is known. Here, we applied a novel genome-wide approach to identify mRNA regions that are bound by yeast methionine aminoacyl tRNA^Met^ synthetase (MetRS). We found a clear enrichment to regions that were previously described to contain pseudouridine (Ψ). Follow-up *in vitro* and *in vivo* analyses on a prime target (position 1074 within *YEF3* mRNA) demonstrated the importance of pseudouridine for MetRS binding. Furthermore, polysomal and protein analyses revealed that Ψ1074 mediates translation. Modification of this site occurs presumably by Pus6, a pseudouridine synthetase known to modify MetRS cognate tRNA. Consistently, the deletion of Pus6 leads to a decrease in MetRS association with both tRNA^Met^ and *YEF3* mRNA. Furthermore, while global protein synthesis decreases in *pus6Δ*, translation of *YEF3* increases. Together, our data imply that Pus6 ‘writes’ modifications on tRNA and mRNA, and both types of RNAs are ‘read’ by MetRS for translation regulation purposes. This represents a novel integrated path for writing and reading modifications on both tRNA and mRNA, which may lead to coordination between global and gene-specific translational responses.

## Graphical Abstract

**Graphical Abstract:**
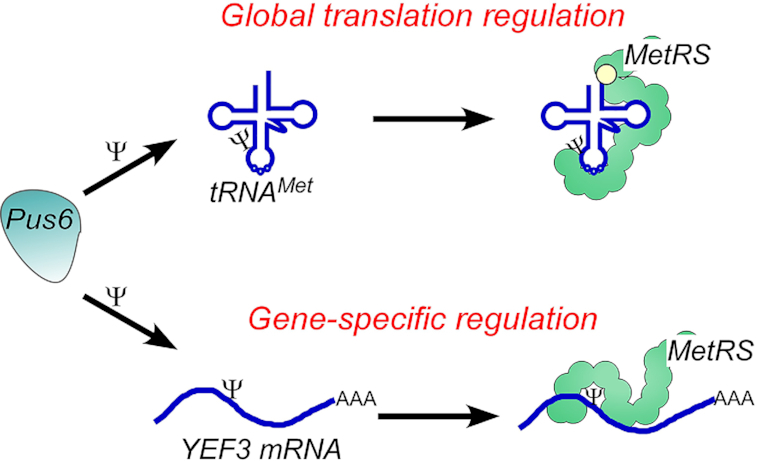
Pseudouridylation by Pus6 enhances MetRS binding of tRNA and mRNA, to confer global and gene-specific translation regulation, respectively.

## INTRODUCTION

Over the past decades, >100 post-transcriptional RNA modifications have been identified in all kingdoms of life (1). One of the most common RNA modifications in living cells is pseudouridine (Ψ) (2,3). The isomerization of uridine to pseudouridine has a similar base pairing as uridine, yet it enables stabilization of RNA structure by an extra hydrogen bond (4,5). In yeast, non-coding RNAs such as rRNA, snoRNA, snRNA and tRNA are highly enriched with pseudouridine at dozens of specific positions (6,7).

The isomerization of pseudouridine is catalyzed by two groups of enzymes (‘writers’). The first group, operating as an RNP complex, uses guide-RNA-based pseudouridine isomerization in which the essential Cbf5 enzyme is guided by H/ACA box snoRNA to its target rRNA sites (7–9). The second pseudouridine synthase (PUS) group is the stand-alone enzyme family that catalyzes the reaction without additional proteins or RNAs (10). Pseudouridine isomerization of cytosolic and mitochondrial tRNAs (11–16) is catalyzed by these enzymes.

Recently, high-throughput pseudouridine-seq studies revealed hundreds of pseudouridine sites in 509 different mRNAs. Most of this mRNA pseudouridylation activity was catalyzed by the stand-alone tRNA PUS family. Moreover, those studies indicated that pseudouridylation may be regulated in response to environmental changes (17–19). While *in vitro* analysis revealed an impact on translation (20), the *in vivo* impact of this post-transcriptional modification on mRNA activity remains largely unknown.

Modifications in RNA molecules usually serve as recognition sites to proteins (‘readers’) that exert a regulatory function. To date, the only identified pseudouridine reader is the yeast Prp5 RNA helicase. Prp5 interacts with Ψ42 and Ψ44 within U2 snRNA and promotes pre-mRNA splicing (21). Surprisingly, no mRNA or tRNA pseudouridine readers have been identified thus far.

The family of aminoacyl tRNA synthetases (aaRS) sustains extensive interactions with pseudouridylated RNAs (i.e. tRNAs). aaRSs identify their cognate tRNA and catalyze the charging reaction of a specific amino acid at the tRNA 3′ end (22). The interaction of a specific tRNA with its cognate aaRS depends on unique tRNA identity elements. These elements are located mainly at the acceptor stem and the anticodon stem–loop (23–25). tRNA identity elements are also a hotspot of RNA modifications. These post-transcriptional RNA modifications can change the tRNA structure and may play a central role in tRNA–aaRS interactions (26). However, the *in vivo* impact of tRNA pseudouridylation on aaRS binding is not known. The association of aaRSs with RNA is not restricted to tRNAs (27,28). Recent interactome studies revealed that aaRSs interact with polyA RNA, likely mRNAs (29–31). Various anticodon-like elements within mRNA appeared key for aaRS-mRNA interaction and regulation (32–34); however, a role for mRNA modification is unknown.

Herein, we performed an *in vivo* binding-site mapping for *Saccharomyces cerevisiae* MetRS. Comparing these data to previously published pseudouridine-seq data revealed that MetRS preferentially binds Ψ-enriched regions. We revealed that Ψ at position 1074 of *YEF3* mRNA and Ψ31 of elongator tRNA^Met^ are important for MetRS binding. The former interaction regulates *YEF3* protein synthesis while the latter has a global effect on translation. Furthermore, our data suggest that both modifications are made by Pus6, suggesting a pivotal role for this enzyme in regulating global and gene-specific translation.

## MATERIALS AND METHODS

### Yeast strains and growth conditions

The following yeast strains were used: endogenously TAP-tagged (TAP-His3MX) *MES1* (MetRS) in the following genetic background (ATCC 201388: *MATa his3Δ1 leu2Δ0 met15Δ0 ura3Δ0*) (35); endogenously GFP-tagged (GFP (S65T)-His3MX)-MES1 (MetRS) in the following background (ATCC 201388: *MATa his3Δ1 leu2Δ0 met15Δ0 ura3Δ0*) (36); and endogenously N terminally GFP-tagged Pus6 (YGR169C) in the background of BY4741 as described in (37). Proper fusion and expression of the tags were verified by PCR and western analysis. The knockout of *PUS6* was done in the MetRS-GFP background by homologous recombination using a LEU2 cassette amplified from pRS405 as described in (38). Cells were usually grown in YPD or SCD media (39) at 30°C to mid-logarithmic phase.

### Fragmentation of RNA and ImmunoPrecipitation (fRIP)

MetRS-TAP strain was grown in YPD to mid-logarithmic phase and subjected to cross-linking by the addition of formaldehyde (0.1% final concentration) for 10 min at room temperature. Cross-linking was terminated with 0.125 M glycine for 3 min, and cells were lysed in Buffer B (20 mM Tris–HCl [pH 7.5], 140 mM NaCl, 0.1% NP40, 0.1% SDS, 0.5 mM EDTA, 1 mM DTT, 2 mM PMSF, 10 μg/ml Leupeptin, 14 μg/ml Pepstatin, 0.02 U/μl RQ1 RNase-free DNase [Promega], 0.24 U/μl RiboLock RNase Inhibitor [Thermo Scientific]). Lysate was cleared by centrifugation for 10 min at 10,600 g at 4°C and fragmented by 0.2 U/μl RNase I (LifeTech, AM2295) for 4 min at 37°C with gentle shaking. For background cleavage pattern, 10% of the cleaved lysate was set aside and RNA was subjected to phenol:chloroform extraction (‘Input’ sample). The remaining 90% of the cleaved lysate was loaded on IgG Sepharose beads for 2 h at 4°C and washed four times in Buffer C (20 mM Tris–HCl [pH 7.5], 1 M NaCl, 0.5% NP40, 0.1% SDS, 0.5 mM EDTA, 0.5 mM DTT, 0.01 U/μl RiboLock RNase Inhibitor [Thermo Scientific]). MetRS was eluted by cleavage with 80 U of TEV (Invitrogen 12575-015) for 2 hr at 16°C in TEV buffer. Crosslinking was reversed by heating at 65°C for 16 h in reverse cross-linking buffer (50 mM Tris–HCl [pH 7.5], 5 mM EDTA, 10 mM DTT, 1% SDS, 0.1 U/μl RiboLock RNase Inhibitor [Thermo Scientific]) and RNA was precipitated following phenol:chloroform extraction (‘IP’ sample).

### RNA-binding proteins ImmunoPrecipitation and qPCR (RIP-qPCR)

RIP-qPCR analysis was done as described in (33). Briefly, for mRNA RT-qPCR, RNA was reverse-transcribed using a high-capacity cDNA reverse transcription kit (Maxima) according to the manufacturer's instructions. Gene-specific levels were determined in a 25 μl reaction volume in triplicate with a Power SYBR Green PCR Master Mix^®^ (Applied Biosystems) two-step RT-PCR method following the manufacturer's instructions using primers for the indicated genes (Supplementary Table S1). All qPCR reactions used the following parameters: 50°C for 2 min, 95°C for 10 min, and then 30 s at 95°C and 1 min at 60°C for 40 cycles. Results were analyzed with Applied Biosystems 7500 Real-Time PCR Software v2.0.6. Fold change was calculated using either 2^−(ΔCt)^ or 2^−(ΔΔCt)^.

### RNA-seq analyses

RNA samples were subjected to RNA-seq at the Technion Genome Center. Libraries from the Input and Bound fRIP samples were prepared using a TruSeq RNA Library Prep Kit v2 (Illumina, CA, USA) according to the manufacturer's instructions. Ribo-Zero rRNA Removal Kit was applied according to the manufacturer's instructions only for the Input samples. All samples were sequenced on Illumina platform, yielding 10 to 30 million reads per sample. Reads were mapped to the S288c *S. cerevisiae* version R64–2-1 genome using RNA STAR version 2.6.0b-2. Peaks were detected using MACS2 callpeak (40) version 2.1.1.20160309.6 against input sample with essential command attributes - Bandwidth of 70, mfold 5–50 and Minimum FDR (*q*-value) <0.05. Peaks with Irreproducible Discovery Rate (IDR) <0.05 were considered statistically significant peaks. RNA-seq data for all samples can be obtained at the European Nucleotide Archive (ENA) with the Primary Accession PRJEB41037 (sample group ERP124760).

### tRNA quantification

tRNA RT-qPCR was done as described in (35). Briefly, tRNA was reverse-transcribed using a RevertAid First Strand cDNA Synthesis Kit (Thermo Fisher Scientific). Reaction temperature was elevated to 60°C, and reverse-transcription elongation was extended to 30 min to efficiently overcome tRNA modification and secondary structure. tRNA-specific levels were determined in a 25 μl reaction volume in triplicate with a Power SYBR Green PCR Master Mix^®^ (Applied Biosystems) following the manufacturer's instructions using primers for the indicated tRNA as described in (35). Results were analyzed with Applied Biosystems 7500 Real-Time PCR Softwarev2.0.6. Fold change was calculated using 2^−(ΔCt)^. tRNA RT-qPCR products were cloned into a pGEM system and subjected to Sanger sequencing to validate amplification of the proper tRNA. All primers are listed in Supplementary Table S1.

### MetRS purification

Rosetta™ 2 bacteria carrying the pMALc2 vector with MBP-MetRS were grown to OD_600_ of 0.5, induced by 0.3 mM IPTG for 3.5 h at 30°C and harvested. Cells were lysed with lysis buffer (250 mM Tris–HCl pH 8.0, 100 mM NaCl, 0.25% Tween20, 10 mM β-mercaptoethanol, 5% glycerol, 0.1 mM PMSF, 0.5 μg/ml Leupeptin, 0.7 μg/ml Pepstatin), lysates were cleared by centrifugation for 15 min at 10 000 × g at 4°C and supernatant was loaded on 500 μl of amylose beads (NEB, E8021S). Tubes were placed on rotation at 4°C for 3 h. Beads and lysate were then poured on a Poly-Prep^®^ Chromatography Column (BioRad). Beads were washed with 15 mL ice-cold washing buffer (30 mM Tris–HCl pH 8.0, 300 mM NaCl, 1 mM β-mercaptoethanol, 5% glycerol). Elution was performed using ice-cold elution buffer (30 mM Tris–HCl pH 8.0, 100 mM NaCl, 1 mM β-mercaptoethanol, 5% glycerol, 1% maltose).

### UV crosslinking of RNA probe

RNA oligonucleotides (GAUUUAAGA or GAUUΨAAGA [chemically synthesized by Dharmacon]) were 5′ labeled by T4 Polynucleotide Kinase (PNK) and γ-^32^P-ATP. Purified MetRS protein (10 ng) was preincubated in binding buffer (10 mM HEPES pH 7.4, 140 mM KCl, 10 mM NaCl, 1 mM MgCl_2_, 0.01% NP-40, 5% glycerol) for 15 min at 25°C. Radiolabeled RNA probes (1 nM) was added and incubated for another 15 min at 25°C. Binding reactions were then irradiated with UV 254 nm (50–300 mJ/cm^2^) on a parafilm sheet on ice. For competition assays, 1 ng of yeast tRNA (R8759 Sigma-Aldrich) was added before crosslinking. Samples were then resolved on 8% PAGE. Gels were dried and exposed to a phosphorimager and radioactive signals were quantified by ImageJ.

### CRISPR/Cas9 point mutagenesis

Point mutations were introduced using the single-plasmid CRISPR/Cas9 protocol (41,42). Briefly, plasmid (bRA66 backbone (Addgene #100952)) expressing guide RNA recognizing the mutation region was transformed into MetRS-GFP strain together with the double-stranded 80 bp DNA fragment that contains the desired mutation at its center (Supplementary Table S1). Transformants were grown on YP-Gal Hygromycin (200 μg/ml) plate to induce Cas9 expression. Three viable clones from the transformation with the 80 bp fragment were isolated and the modification region was sequenced; all included only a single change, T1074C.

### ^35^S methionine pulse labeling

Cells (10 ml) were grown to mid-log phase in synthetic complete (SCD) medium, washed and resuspended in 10 ml SC without methionine. ^35^S methionine (NEG709A005MC, PerkinElmer) was added and growth was continued for another 30 min. The translation was quenched by the addition of cycloheximide (100 μg/ml final concentration). Proteins were extracted and resolved on 10% PAGE. Gels were exposed to a phosphorimager and radioactive signals were quantified by ImageJ.

### Polysomal analysis

Polysomal analysis was done as described in (43). Briefly, 100 ml cells grown in YPD to mid-logarithmic stage were harvested and lysed by a bead beater in 0.4 ml of lysis buffer (20 mM Tris–HCl at pH 7.4, 140 mM KCl, 1.5 mM MgCl_2_, 0.5 mM dithiothreitol, 100 μg/ml cycloheximide, 1 mg/ml heparin, 1% Triton X-100). The lysate was cleared and centrifuged for 15 min at 9000 g at 4°C and the supernatant was loaded onto a 12 ml 10–50% linear sucrose gradient. Gradients were centrifuged in a SW41 rotor (Beckman) at 35 000 rpm for 160 min and polysomal profiles were determined by monitoring RNA absorbance at 254 nm with ISCO UA-6. Polysomal association was calculated by dividing the polysomal area by the area from the beginning of the 40S to the end of the graph. The entire gradient was fractionated to fractions of similar volume. RNA was extracted from each fraction by the addition of an equal volume of 8 M guanidinium HCl and two volumes of 100% ethanol, overnight incubation at –20°C and centrifugation at 13 000 rpm for 30 min. Pellets were washed with 80% cold ethanol, and usually half of the sample was subjected to northern blot analysis with the indicated probes.

## RESULTS

### Fragmented RIP-seq (fRIP-seq) protocol for mapping MetRS-bound RNA regions

**Figure 1.**
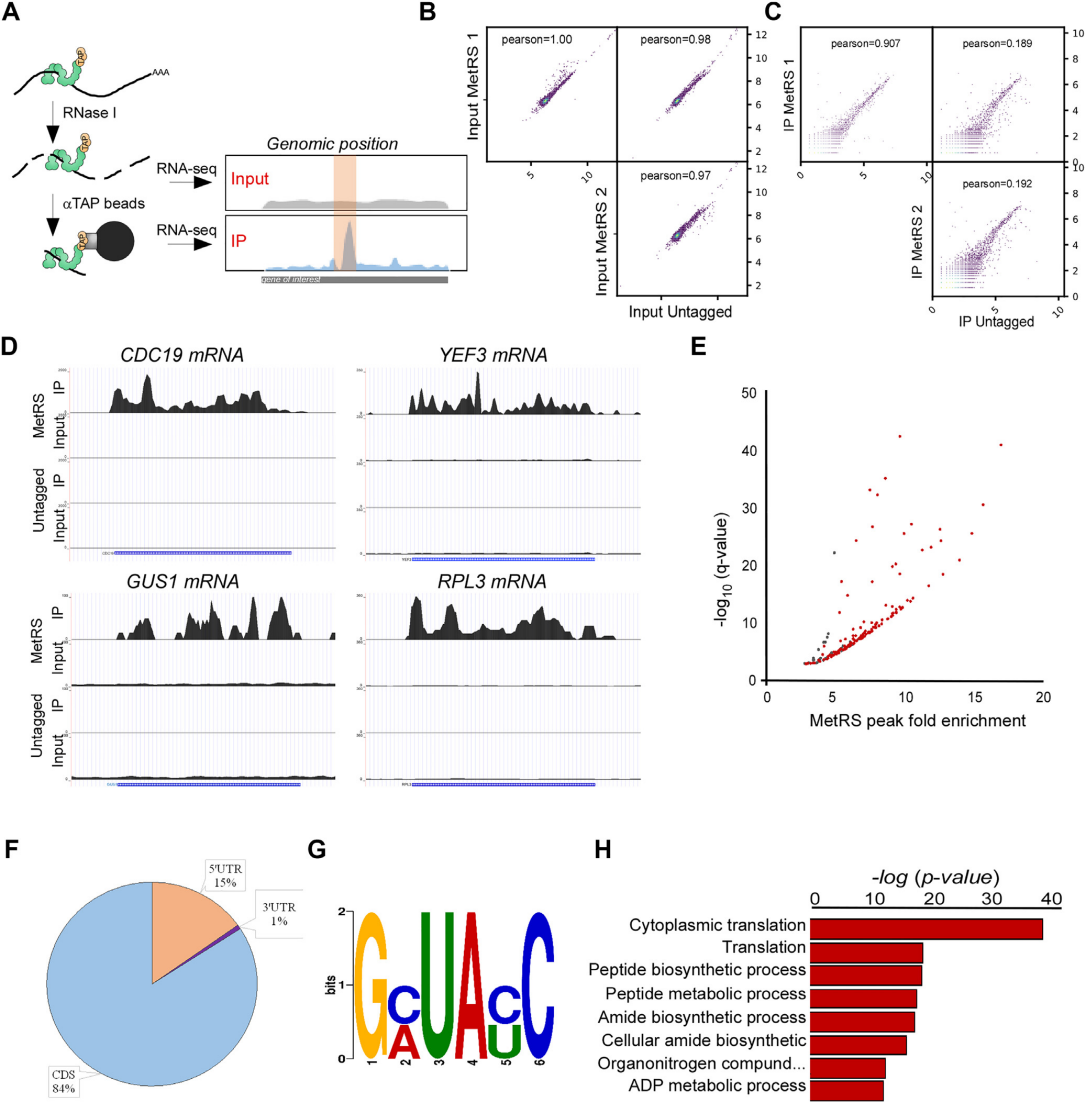
fRIP-seq protocol to identify RBP’s binding regions. (**A**) Scheme of the fRIP-seq protocol. Cell lysates of TAP-tagged MetRS strain are subjected to mild RNase I treatment. Fragmented lysate was mixed with IgG Sepharose beads to isolate the MetRS-TAP-bound RNA. Input and immunoprecipitated (IP) fragments were subjected to RNA-seq. Peak calling algorithm (40) is used to define regions that are enriched in the IP compared to the Untagged control fragments. (**B**) fRIP-seq Input RNA samples from two biologically independent MetRS-TAP cultures and from untagged parental strain were subjected to RNA-seq. Scatter plots present the pairwise comparisons of sequencing reads. Pearson correlation coefficient is shown in each box. (**C**) fRIP-seq for IP samples, collected and analyzed as in B. (**D**) Examples of fRIP-seq read maps of IP and Input data from MetRS-TAP and untagged strains. Y axis to the left of every map indicates the number of reads (note that scales differ between genes). Schematic depiction of the gene is indicated below each map. (**E**) Assignment of significant peaks. Scatter plot indicates the fold-enrichment (compared to Untagged control) for each peak defined by MACS2 callpeak algorithm (40) vs. the significance (as log_10_ of the *q*-value). Red-marked dots indicate peaks that are defined as significant (Irreproducible Discovery Rate (IDR) < 0.05). (**F**) Pie chart indicates the position of significant peaks along detected genes. (**G**) MetRS-bound peaks were analyzed for an enriched sequence motif by DREME (59). (**H**) MetRS-bound peaks were analyzed for enriched process terms by SGD GO Term Finder Version 0.86. The most significant terms (corrected *P*-value <9.49E–13) are presented.

Our previous RNA-binding protein ImmunoPrecipitation (RIP-seq) study revealed a subset of mRNAs that are highly associated with yeast methionine aminoacyl synthetase (MetRS) (33). To narrow down RNA regions that MetRS is associated with, we established a fragmentation of RNA and RIP-seq (fRIP-seq) protocol. In fRIP-seq, cellular lysate of a TAP-tagged MetRS strain (or, for that matter, any other RNA-binding protein) is subjected to a mild RNase I treatment prior to RNA-binding protein immunopurification (RIP) with IgG Sepharose beads. Fragmented samples from either before RIP (Input) or those eluted from the beads by TEV cleavage (IP) were analyzed by next-generation sequencing (Figure 1A). The genomic position of Input and IP fragments was compared and enriched positions (‘peaks’) were defined. While we applied this approach herein to MetRS-TAP, we note that we successfully applied it to GFP-tagged proteins using Chromotek GFP-Trap beads (Levi O. *et al.*, in preparation).

The mild fragmentation in fRIP generates RNA fragments of ∼200 nts, thereby defining the resolution of the approach (Supplementary Figure S1A). About 25% of tagged MetRS proteins were detected in the elution sample, indicating the efficiency of MetRS isolation (Supplementary Figure S1B and C). Western analysis for Hxk1 control protein did not detect any signal at the bound sample, indicating the specificity of isolation. Furthermore, silver staining of all proteins in the bound sample detected only marginal levels of proteins other than MetRS, and all were detected in the control purification of an untagged strain (Supplementary Figure S1C). Overall, under these conditions, MetRS appears to be efficiently and specifically isolated.

RNA samples from two biological repeats of fragmented cell lysate (Input) and the IP sample were sequenced. A similar scheme was applied to the parental untagged strain to control for non-specific binding. A high correlation is apparent between the Input signals of the two MetRS repeats and the untagged strain (Figure 1B). Thus, the TAP-tagging of MetRS does not lead to a general effect on gene expression. Analysis of the IP samples revealed that while both biological repeats of MetRS-TAP were very similar (Pearson correlation of 0.907), both were significantly different from the untagged control IP (Figure 1C). Thus, the fRIP-seq protocol is very reproducible, attested by the high correlation between biological repeats, and highly specific, apparent from the low correlation with the untagged strain. Figure 1D presents read maps of four representative mRNAs. The high efficiency and specificity of binding is apparent upon comparison to the Input sample and/or the Untagged strain data.

Applying a peak calling algorithm (40) to define mRNA regions that are significantly higher compared to the overall background of the mRNA and reproducible in both biological repeats, identified 145 such regions scattered within 131 mRNAs (Figure 1E, Supplementary Table S2). Interestingly, these regions are mostly localized within the coding region of the mRNA (Figure 1F). DREME sequence alignment (44) identified several significant motifs, all containing UA dinucleotide, with GNUANC being the most significant (Figure 1G, Supplementary Figure S1D); we did not find any resemblance to sequences within canonical targets of MetRS (i.e. tRNA^Met^) (45). GO term analysis revealed that binding is significantly enriched to mRNAs encoding translation factors (GO: 0002181) (Figure 1H, Supplementary Table S3). Altogether, these data suggest that MetRS regulates expression of translation factors through binding to their coding regions.

### MetRS is a pseudouridine reader

**Figure 2.**
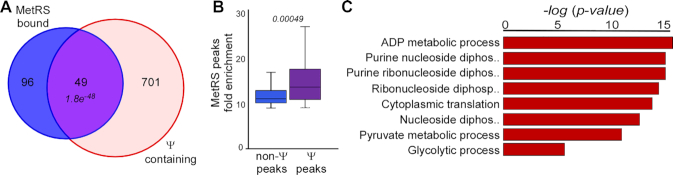
MetRS binds Ψ-enriched regions. (**A**) Venn diagram was generated from peaks identified as bound by the MetRS fRIP-seq analyses and specific mRNA ribonucleotide that was found to contain pseudouridine by Ψ-mapping studies (17–19). *P-value* was calculated by the Hypergeometric test. (**B**) MetRS bound peaks were separated into those that do not contain Ψ (non-Ψ) and those that do. Box plot presents the fold enrichment in binding compared to untagged control. *P* -value was calculated by the two-tailed *t*-test. (**C**) GO terms processes analysis was done to Ψ-containing peaks genes that are bound by MetRS using SGD GO Term Finder Version 0.86.

The canonical function of MetRS is charging of initiator and elongator tRNA^Met^ with methionine. These tRNAs are known to be modified extensively, in particular at uridines that are modified into pseudouridine (Ψ). Therefore, we examined whether MetRS-bound RNAs are enriched with Ψ. Three independent pseudouridine-seq studies were previously published (17–19). While these datasets are likely incomplete, as apparent from the low overlap in their hits (46,47), altogether they report on 750 sites of Ψ within *S. cerevisiae* transcriptome. We compared these sites with the MetRS-bound fragments detected here, and a significant number (49 of 145, *P*-value 1.8e^−^^48^) appeared to overlap (Figure 2A, Supplementary Table S4). Moreover, the peaks that contain a Ψ appear to be better bound by MetRS compared to the 96 not reported to be pseudouridylated (Figure 2B). Thus, pseudouridine enhances MetRS binding to its target regions. GO term analysis for the 49 peaks that contain pseudouridine recaptured the enrichment to mRNAs-encoding cytosolic translation factors (Figure 2C, Supplementary Table S3), suggesting that regulation of this family occurs through Ψ.

### Ψ1074 of *YEF3* is important for MetRS binding

**Figure 3.**
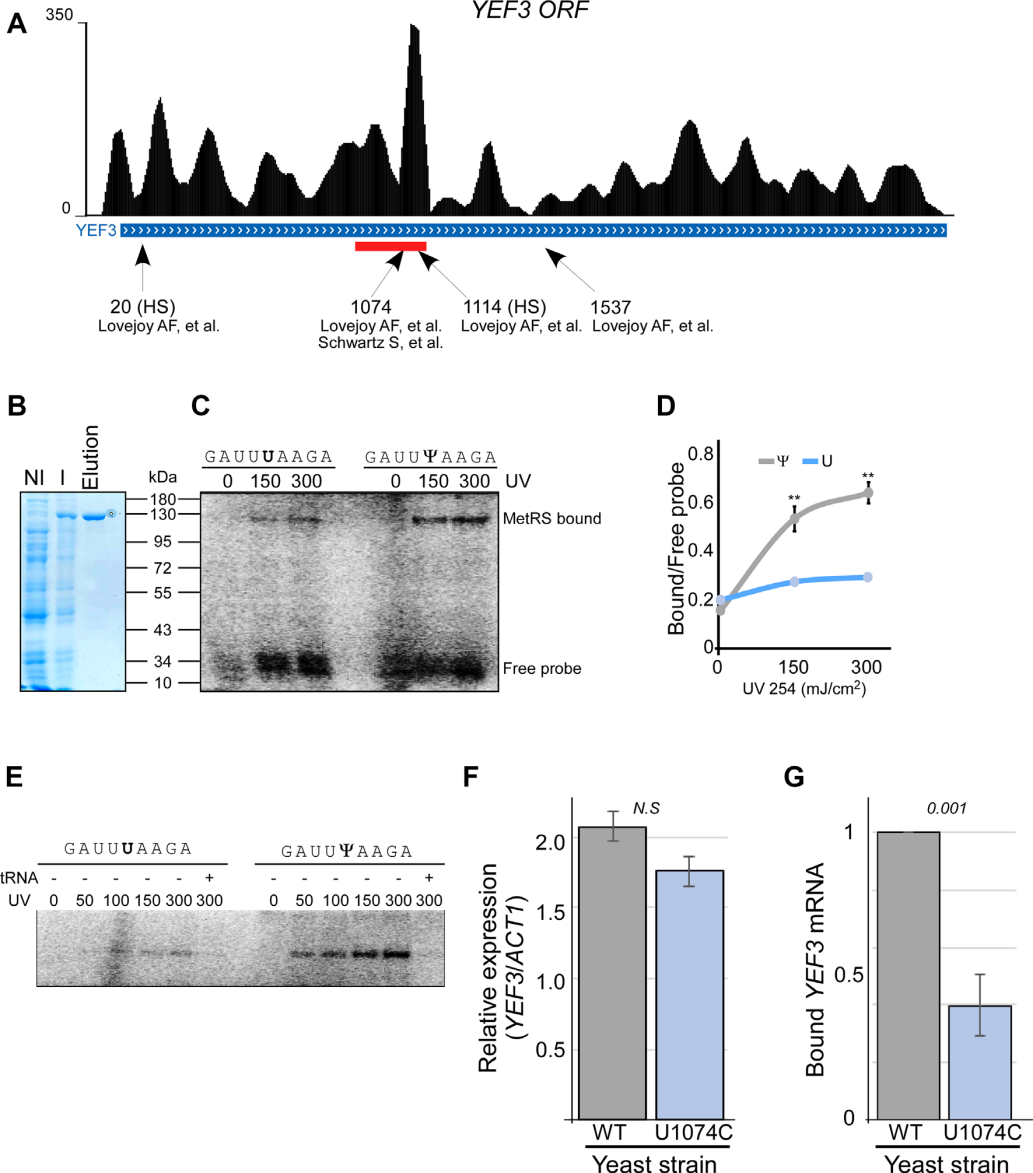
MetRS binds *YEF3* mRNA at Ψ1074. (**A**) Genome browser map showing fRIP-seq coverage along *YEF3* mRNA (UCSC Genome Browser). Red bar under the map indicates the MetRS binding region, as identified by the MACS2 callpeak (40). Arrows point to Ψ positions revealed by (18,19) along *YEF3* ORF. HS indicates sites apparent upon heat shock. (**B**) Coomassie blue staining of MetRS purification steps. Noninduced lane (NI) contains a protein sample from bacteria transformed with MBP-MetRS plasmid before induction. Input (I) contains a protein sample from bacteria after expression induction. Elution includes the protein sample eluted from the MBP column. (**C**) Labeled 9-mer oligonucleotide (identical to the sequence surrounding Ψ1074), either with or without Ψ at its center, was UV-crosslinked to the indicated extent with purified MetRS and resolved on PAGE. Gel was dried and exposed to a phosphorimager. (**D**) Quantification of the MetRS-bound oligo to free oligo ratio from two independent crosslinking experiments. ** indicates *P* -value ≤0.01 for the difference between Ψ and U containing oligos at each UV exposure. Note that error bars for the U oligo are smaller than the data points. (**E**) UV crosslinking was done at the indicted amounts. Competitor cold yeast tRNA was added prior to crosslinking where indicated. (**F**) Point mutation was introduced by CRISPR/Cas9 into the genomic site of *YEF3*, replacing the encoded U1074 with C. Steady-state levels of wildtype (WT) and mutant (U1074C) *YEF3* mRNA were quantified by RT-qPCR analysis from three independent biological repeats, each with three technical repeats, normalized to *ACT1* mRNA levels. (**G**) MetRS-GFP expressing yeast strains, co-expressing endogenously either WT or U1074C *YEF3* mRNA, were subjected to RIP. Amounts of bound *YEF3* mRNAs were quantified by RT-qPCR analysis and normalized to their expression levels. The histogram presents the quantification of two independent biological repeats, each with three technical repeats. *P*-value was calculated by the dependent samples one-tailed t-test.

MetRS appeared to significantly bind a region around nt 1000 of the mRNA-encoding eEF3 elongation factor (*YEF3*) (Figure 3A, red bar). This region was reported by two independent studies to include a Ψ site at position 1074 downstream to the start codon (chrXII:637854) (18,19). Nucleotide 1074 is a wobble position of a UUU codon (Phe amino acid). Position 1075 is occupied by an A, thus generating a UA dinucleotide, in line with the core apparent by computational predictions (Supplementary Figure S1D). To examine the possibility that MetRS binds this site, we expressed *S. cerevisiae* MetRS protein in bacteria and purified it through MBP column (Figure 3B). Purified protein was mixed and crosslinked with labeled 9-mer oligos identical to the 1074 region with either Ψ or U at its center (Figure 3C). A 2-fold higher association was observed in the Ψ-containing 9-mer compared to the mutant (Figure 3D). Importantly, the addition of a competitor cold tRNA abolished binding to either oligo (Figure 3E). We note that attempts to derive exact disassociation constant (*K*_D_) using EMSA were futile, probably because affinity to the 9-mer was too low (not shown). Thus, the optimal MetRS binding site is probably longer.

To examine the significance of Ψ at position 1074 (Ψ1074) *in vivo*, we replaced the thymidine (T) in this genomic position with cytosine (C), thereby eliminating pseudouridylation, yet maintaining the same amino acid (both UUU and UUC encode Phe). The mutation was introduced in a seamless manner by CRISPR/Cas9, without the insertion of any selection marker or any other change in the *YEF3* gene (42). Analysis of steady-state *YEF3* mRNA levels revealed a small and insignificant change in the U1074C mRNA levels (Figure 3F). Significantly, however, RIP followed by RT-qPCR to cells expressing MetRS-GFP with either normal or U1074C *YEF3* mRNA revealed a strong reduction in MetRS association with U1074C mutant compared to the WT transcript (Figure 3G). This demonstrates that Ψ1074 is important for MetRS association with *YEF3* mRNA *in vivo*.

### Ψ1074 confers a translation regulatory role

**Figure 4.**
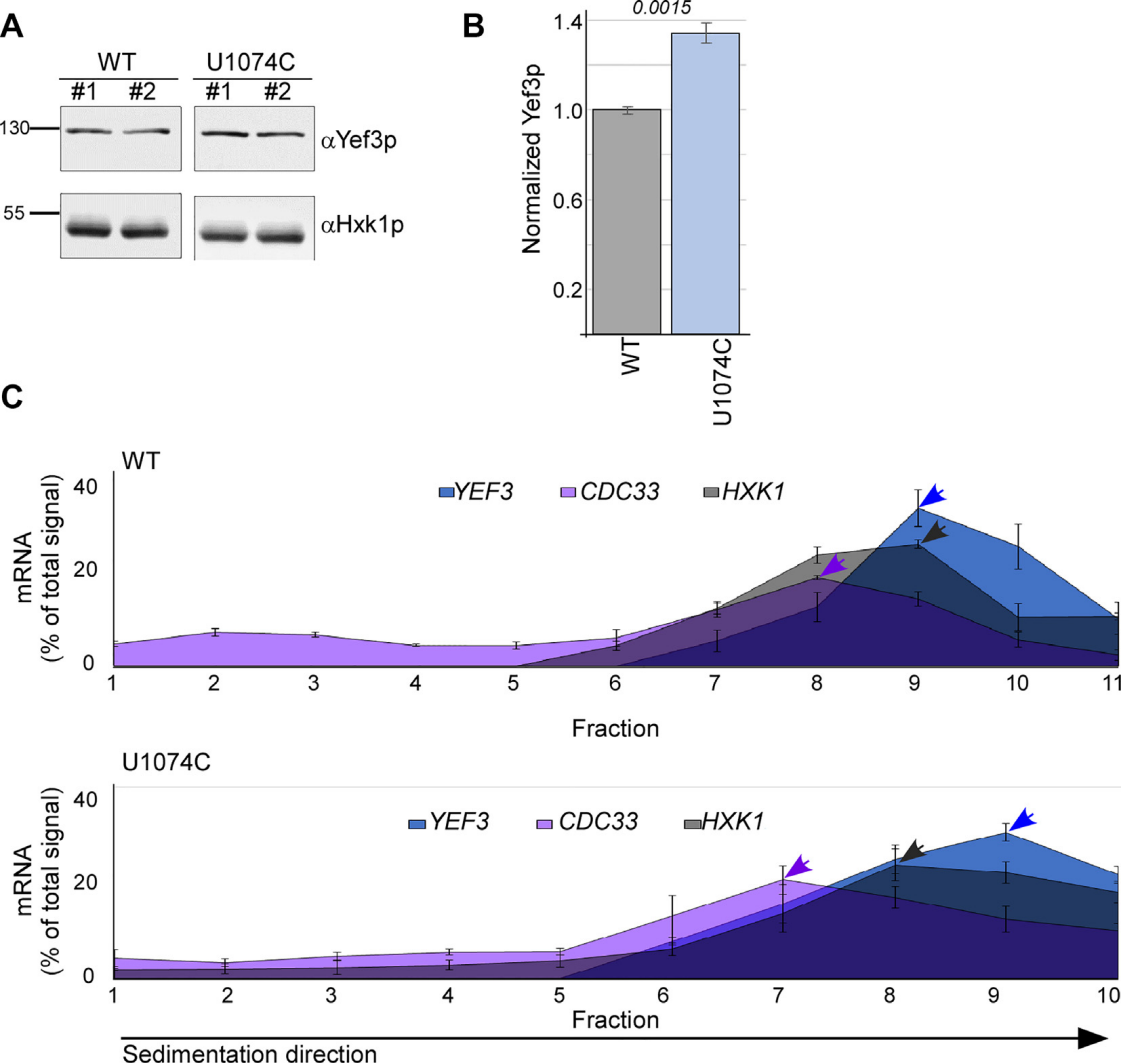
Ψ1074 imposes a translation regulatory role. (**A**) Cells expressing either WT or U1074C transcripts were subjected to western analysis with antibodies recognizing Yef3p or Hxk1p. Two independent protein extractions are presented. Signals are from the same blot from which irrelevant lanes were cropped. (**B**) Quantification of the western blots for Yef3p (normalized to the Hxk1p) from three independent biological repeats. Error bars are SEM, and *P*-value was calculated by the dependent samples one-tailed *t*-test. (**C**) Polysomal separations on sucrose gradients for WT and U1074C cells were analyzed by northern analysis for the indicated transcripts. Graphs present the northern signals quantifications from three independent biological repeats (actual blots are in Supplementary Figure S3). Arrowheads point to the fraction with the highest signal (‘peak fraction’) per mRNA. Error bars are SEM of three independent biological repeats. Note that the fraction collector was inadvertently set to collect 10 fractions for U1074 gradients while the WT were separated into 11 fractions. Yet in both cases the entire gradient was collected (gradients’ OD254 profiles are provided in Supplementary Figure S3A).

Protein binding to an mRNA usually serves expression regulation purposes, which can be exerted through changes in mRNA stability or translation. Since we did not detect significant changes in mRNA steady-state levels upon U1074C change (Figure 3F), we tested possible impact on protein synthesis. Western analysis revealed a clear increase in Yef3 protein levels upon U1074C point mutation (Figure 4A and B). We note that U1074C mutation leads to a silent codon change from UUU to UUC (both code for Phe), with UUC having a lower codon usage than UUU (18.4 versus 26.1) (48). Since lower usage is associated with lower translation (49), it is unlikely to explain the increased protein amounts we observe. Furthermore, both codons are recognized by the same tRNA (tRNA^GAA^) as no tRNA^AAA^ exist *S. cerevisiae* (45); hence, differences in cognate tRNA abundance also cannot explain the increased protein levels.

To substantiate the link to translation, polysome profiling through sucrose gradients was done to both strains. While ribosomal association of unmutated transcripts (i.e. *HXK1* and *CDC33*) appeared not to change, and possibly even decreased in the U1074C strain, the ribosomal association of the U1074C transcript appeared to increase (i.e. shifted to the right) (Figure 4C, Supplementary Figure S3B). Thus, Ψ1074 mediates *YEF3* translation.

### Pus6 affects MetRS binding to tRNA

**Figure 5.**
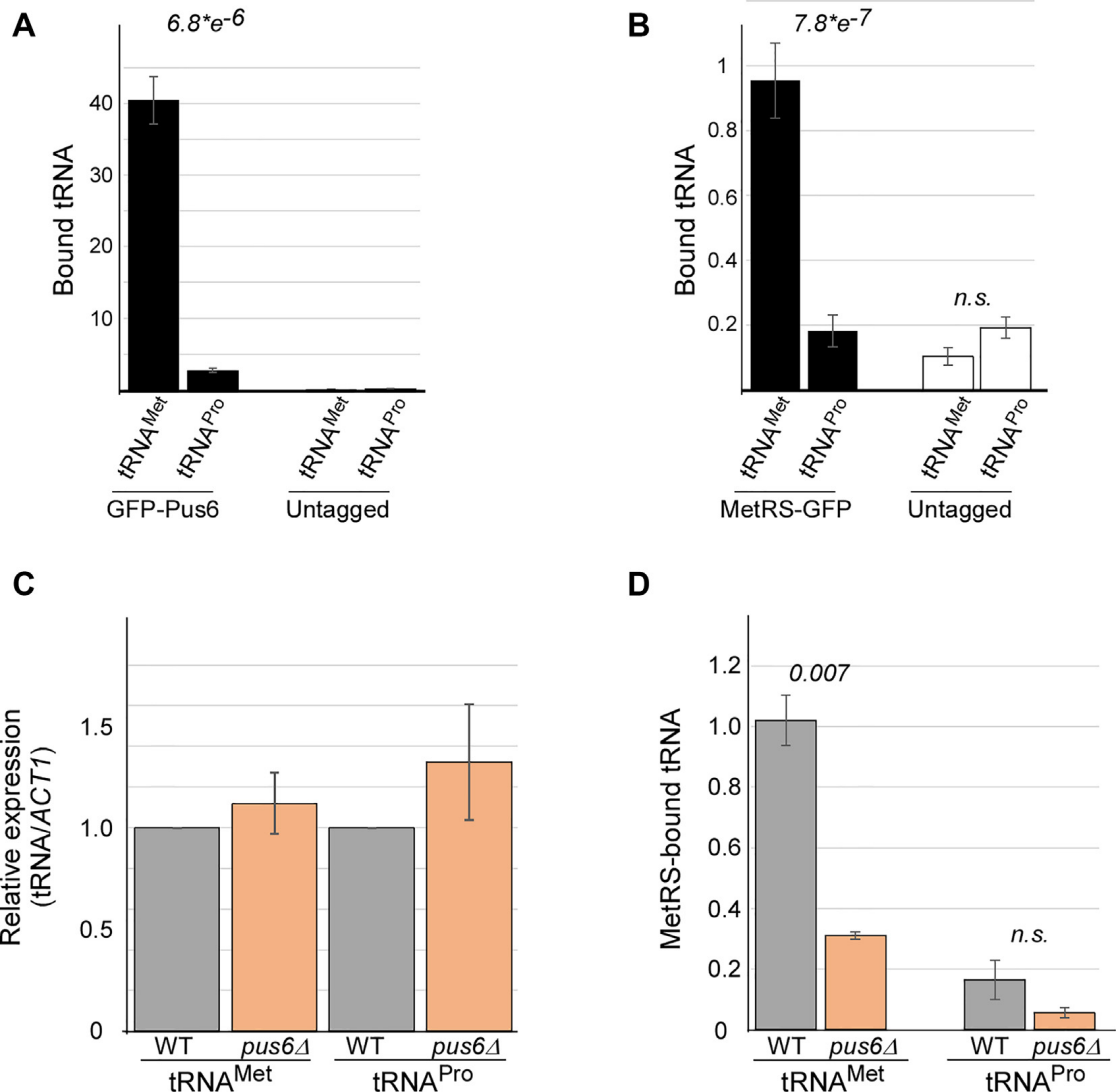
Pus6 mediates MetRS binding to elongator tRNA^Met^. (**A**) GFP-tagged Pus6 strain and a control untagged parental strain were subjected to RIP. Indicated tRNAs were quantified by tRNA-adapted RT-qPCR (35) for the Input and IP samples. Numbers on bars are binding efficiency (IP samples normalized to the corresponded Input). Data are from two independent biological repeats. *P*-value was calculated by the dependent samples one-tailed *t*-test. (**B**) GFP-tagged MetRS strain and a control untagged parental strain were subjected to RIP. Indicated tRNAs were quantified by tRNA-adapted RT-qPCR for the Input and IP samples and analyzed as in A. Data are from two independent biological repeats. *P*-value was calculated by the dependent samples one-tailed *t*-test. (**C**) Steady-state levels of the indicated tRNAs from cells either containing (WT) or deleted of Pus6 (*pus6Δ*) were measured by RT-qPCR analysis (35) and normalized to *ACT1*. The histogram presents the quantification of three independent biological repeats. (**D**) GFP-tagged MetRS cells either containing (WT) or deleted of Pus6 (*pus6Δ*) were subjected to RIP and bound tRNA levels were quantified. The histogram presents the quantification of three independent biological repeats. *P*-value was calculated by the dependent samples one-tailed t-test.

Pseudouridine synthetases (PUSs) isomerize U to Ψ among many tRNAs. The Pus6 member of this family has only one cytosolic tRNA target, elongator tRNA^Met^. Furthermore, U31 within elongator tRNA^Met^ is the only known cytosolic target of Pus6 (14). We performed RIP analysis for Pus6 tagged by GFP to examine its tRNA association *in vivo*. Purification efficiency of this protein appears similar to the other GFP-tagged MetRS strains (Supplementary Figure S2). RT-qPCR designed for tRNA detection (35) was used to explore its targets. A strong association with tRNA^Met^ elongator is apparent, more than 10-fold higher than the control, non-target tRNA^Pro^ (Figure 5A). A similar preference was observed for MetRS binding to tRNA^Met^ (Figure 5B). These data provide an *in vivo* support to tRNA binding specificity of Pus6 and MetRS.

We next wished to determine the impact of pseudouridylation by Pus6. The deletion of Pus6 did not have a significant effect on steady-state levels of either tRNA^Met^ or tRNA^Pro^, suggesting that the modification is not important for tRNA stability (Figure 5C). Importantly, however, RIP analysis to MetRS-GFP detected a strong reduction in MetRS binding to elongator tRNA^Met^ in *pus6Δ*, and a smaller and insignificant impact on binding to tRNA^Pro^ (Figure 5D). This is not due to a decrease in MetRS purification upon Pus6 deletion (Supplementary Figure S2). To the best of our knowledge, this is the first time that pseudouridylation of a tRNA is linked to tRNA synthetase binding *in vivo*.

### Pus6 mediates global protein synthesis

**Figure 6.**
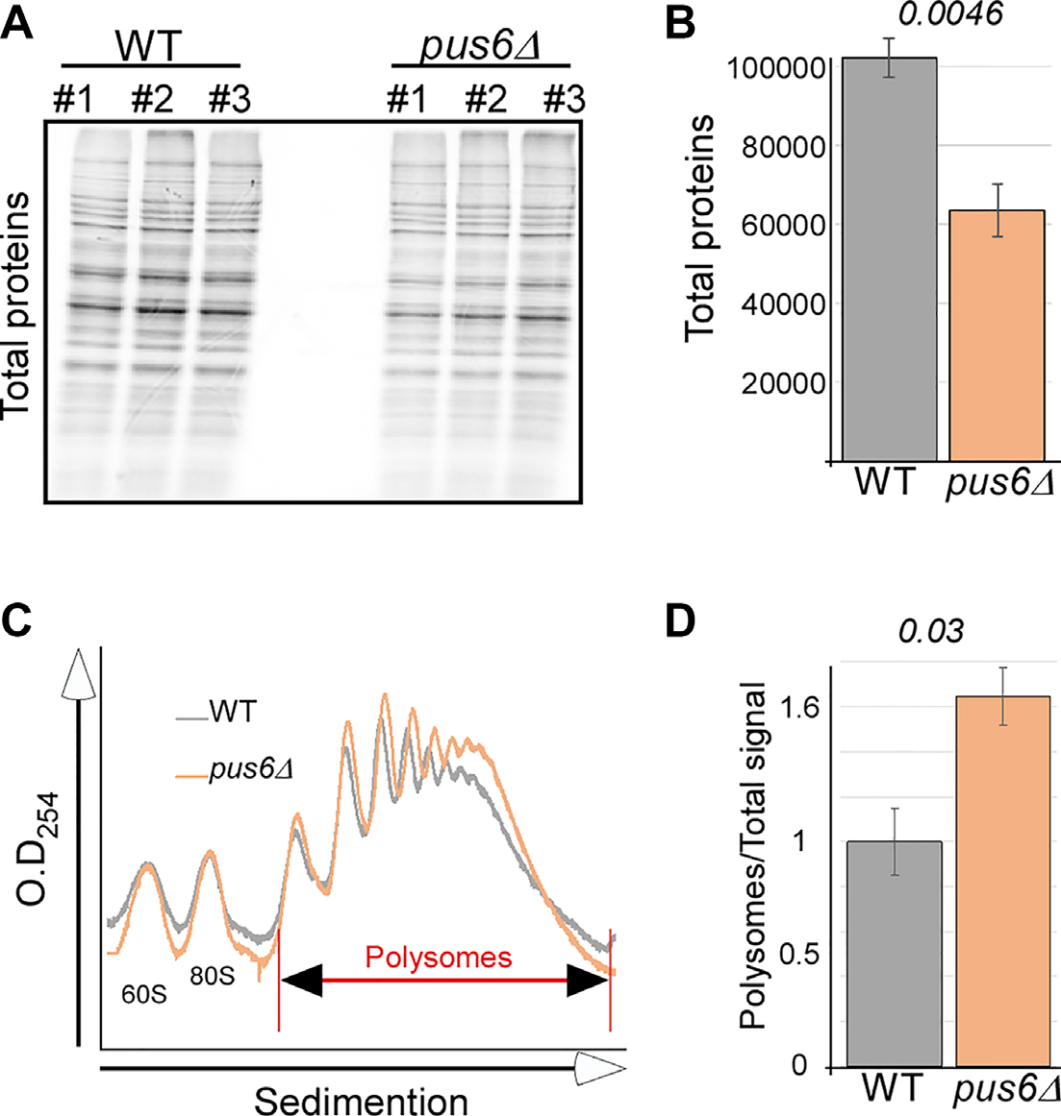
Pus6 deletion affects global translation. (**A**) *pus6Δ* or its isogenic parental strain (WT) were grown to the same logarithmic OD and pulse-labelled with ^35^S-met. Protein samples were collected after 30 min and resolved by SDS-PAGE. Gels were exposed to a phosphorimager. Protein samples from three independent biological repeats (#1 to #3) are presented. (**B**) Quantification of three independent biological repeats of total proteins signal. Error bars represent SEM, and *P*-value was calculated by the dependent samples two-tailed *t*-test. (**C**) WT and *pus6Δ* cells were subjected to polysomal analysis on sucrose gradients. OD254 was monitored throughout the gradient, and the sedimentation position of polysomal complexes (>2 ribosomes) is indicated. (**D**) Quantification of the polysomal fractions signal from three independent biological repeats. Error bars are SEM, *P* value was determined by the dependent samples two-tailed *t*-test. Blots are presented in Supplementary Figure S3C.

The impact of pseudouridylation on tRNA^Met^ binding to MetRS may lead to a broad impact on protein synthesis. To examine this, we performed pulse labeling for all cellular proteins by ^35^S-met labeling of *pus6Δ* and its parental strain (WT) (Figure 6). A clear reduction in their synthesis rates upon Pus6 deletion is observed (Figure 6A and B). Intriguingly, polysomal RNA analysis revealed an increase in OD 254 signal of polysomal fractions in *pus6Δ* cells (Figure 6C and D). This suggests that the lower protein levels are due to slowed translation elongation, leading to accumulation of ribosomes on mRNAs upon Pus6 deletion.

### Pus6 binds and affects *YEF3* translation

**Figure 7.**
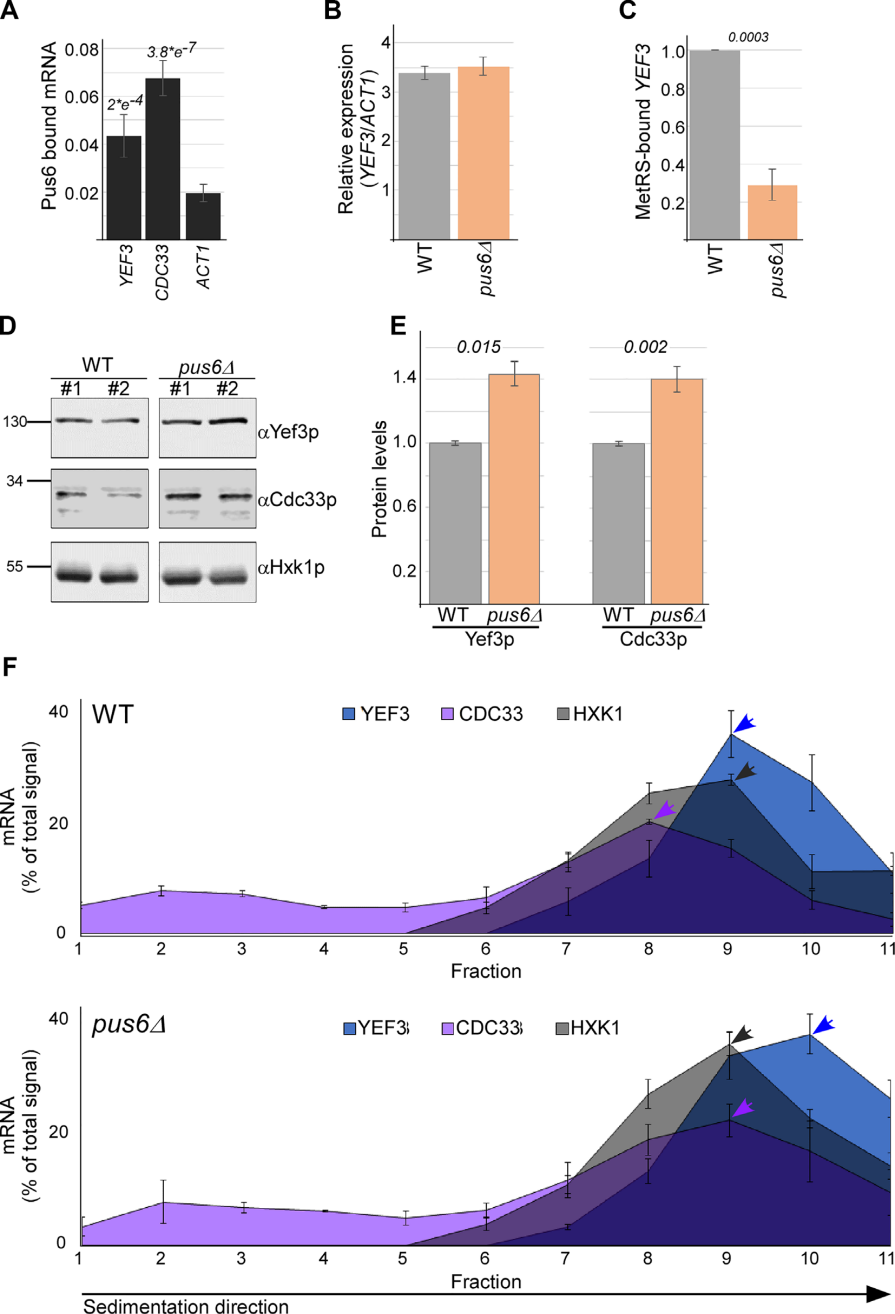
Pus6 regulates translation of pseudouridylated mRNAs. (**A**) GFP-tagged Pus6 cells were subjected to RIP followed by RT-qPCR to the indicated mRNAs. The histogram presents the quantification of two independent biological repeats. *P*-values are relative to *ACT1* mRNA levels and were calculated by the dependent samples one-tailed *t*-test. (**B**) RT-qPCR analysis of *YEF3* mRNA levels normalized to *ACT1* in WT and pus6 deleted cells (*pus6Δ*). The histogram presents the quantification of two independent biological repeats. (**C**) MetRS-GFP expressing cells either containing (WT) or deleted of Pus6 (*pus6Δ*) were subjected to RIP followed by RT-qPCR to *YEF3* mRNA. The histogram presents the quantification of two independent biological repeats, normalized to the signal in the Input sample. *P*-value was calculated by the dependent samples one-tailed t-test. (**D**) Western analysis for the indicated proteins in WT and in *pus6Δ* strains. Samples from two independent protein preparations are presented. Signals are from the same blot, from which irrelevant lanes were cropped. (**E**) Quantification of Yef3p and Cdc33p signals in WT and *pus6Δ* cells normalized to the Hxk1p signal. Results are from three independent biological repeats. Error bars are SEM, and *P*-value was calculated by the dependent samples one-tailed *t*-test. (**F**) WT and *pus6Δ* cells were subjected to polysomal separation followed by northern analysis for *YEF3*, *CDC33* and *HXK1* transcripts. Graphs present the quantification of northern signals within all polysomal fractions. Arrowheads point to the fraction with the highest signal (‘peak fraction’) per mRNA. Results are from three independent biological repeats, error bars are SEM.

Pus6 was previously reported to modify mRNAs (18). Therefore, we tested its possible role in mRNA regulation. *In vivo* binding of Pus6 to mRNAs was assayed by RIP followed by RT-qPCR (Figure 7A). A significantly higher association with *YEF3* than with an mRNA that was not found to be pseudouridylated (*ACT1*) was observed. A strong association was also observed with *CDC33* mRNA (encoding the translation initiation factor eIF4E), supplementing previous *in vitro* pseudouridylation analysis by purified Pus6 on *CDC33* mRNA (18). Furthermore, Pus6 deletion did not affect steady-state *YEF3* mRNA levels (Figure 7B), yet led to a significant reduction in MetRS association with *YEF3* (Figure 7C). Thus, Pus6 is a ‘writer’ of pseudouridine on *YEF3* that is ‘read’ by MetRS.

Concomitant with the decreased MetRS association, analysis of *YEF3* protein levels revealed a clear increase upon Pus6 deletion (Figure 7D and E). A similar increase is observed for *CDC33*, another target of Pus6 (Figure 7D and E). Polysomal analysis to pinpoint the impact to translation revealed no change in the sedimentation of the control *HXK1* mRNA, while both modified mRNAs, *CDC33* and *YEF3*, have a higher polysomal association in *pus6Δ* cells (Figure 7F). Together, these data reveal that Pus6 deletion imposes an activating effect on translation of its target mRNAs, presumably through reduced MetRS binding to Ψ sites.

## DISCUSSION

**Figure 8.**
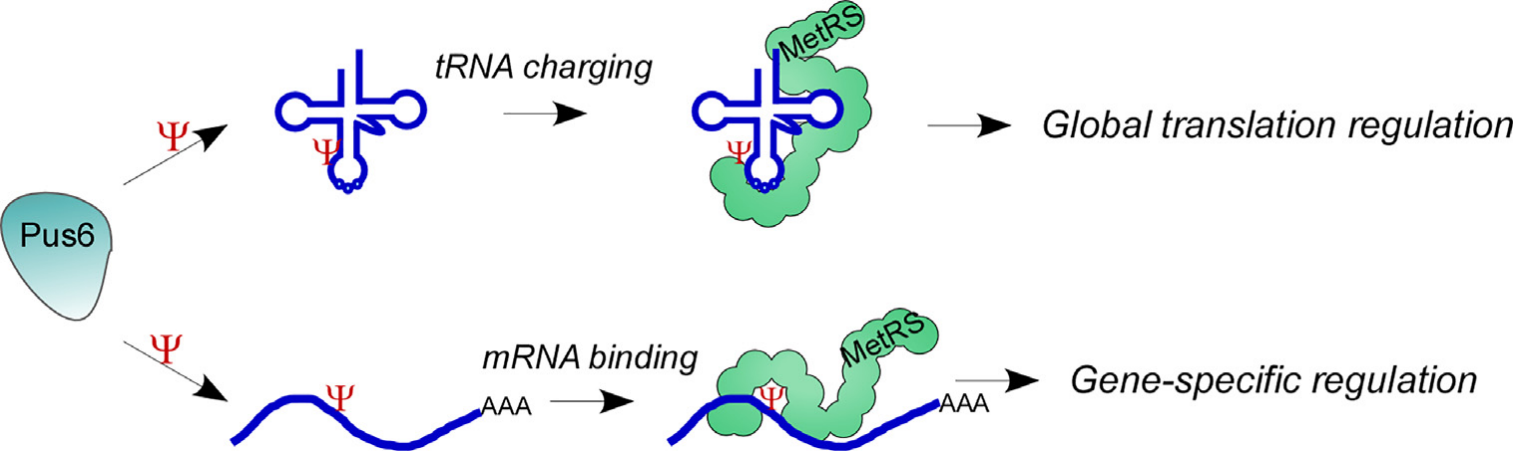
Proposed model for global and gene-specific regulation by Pus6 and MetRS. Pus6 modifies uridines to pseudouridines on both elongator tRNA^Met^ and target mRNAs (e.g. *YEF3*). These modifications enhance MetRS binding on both types of RNA. MetRS binding to tRNA^Met^ presumably affects its charging and thereby has a global impact on translation, while MetRS binding to an mRNA leads to a gene-specific impact.

Functions of aaRS other than tRNA charging are well known and include diverse roles in mRNA regulation (50,51). Interestingly, some of these functions are mediated through recognition of mRNA elements that resemble tRNA elements (24). For example, elements that are similar to anticodons of tRNA^His^ or tRNA^Thr^ were found within mRNA targets of HisRS and ThrRS, respectively, (33,34) and both exert a translation regulatory role. Here, we show for the first time, the involvement of another established tRNA feature, Ψ modification, in mRNA recognition by aaRS followed by translation control. Specifically, we show that MetRS interacts with both tRNA and mRNA through a pseudouridine that is generated by Pus6. Interaction with these two types of RNA offers coordination between tRNA charging and mRNA translation. Our working model (Figure 8) poses that Pus6 modifies elongator tRNA^Met^, and this modification is important for MetRS binding (Figure 5D). The decreased association with MetRS likely leads to a decrease in tRNA^Met^ charging. We speculate that lower levels of charged tRNA^Met^ reduce rates of translation elongation, hence, the increase in polysomal complexes observed in *pus6Δ* cells (Figure 6D). In parallel, Pus6 is a pseudouridine writer on specific mRNA targets (Figure 7A) (17,18). These sites are probably important for MetRS binding to mRNA, therefore upon Pus6 deletion the association of MetRS decreases (Figure 7C). Importantly, we show that Pus6 deletion leads to increased translation of mRNAs that are known to have a pseudouridine (Figure 7D–F). Altogether, we propose that pseudouridines introduced by Pus6 are read by MetRS to exert a global and a gene-specific translation regulation.

What is the regulatory purpose of this pathway? The opposing effects on general and gene-specific translation echo the well-studied yeast amino acid starvation pathway (52). In response to amino acids depletion, GCN2 phosphorylates eIF2α, which leads to sequestration of eIF2B, followed by inhibition of global translation yet increased translation of GCN4 mRNA (53,54). While the mechanistic details of our observation are different, it suggests another path for coordination between global and gene-specific regulation in response to environmental changes. Thus far, we were unable to identify the environmental conditions upon which Pus6 is activated; the obvious predictions of methionine or general amino acid changes did not appear to change Pus6 levels (data not shown). Nevertheless, pseudouridylation by Pus6 appears as a new means to coordinate between tRNA and mRNA functions and global and gene-specific translation regulation. Since pseudouridine is among the most abundant modifications in RNA, we expect that regulation will be implicated on many transcripts. Furthermore, a significant fraction of mRNAs that include Ψ and bound by MetRS encode components of the translation machinery. These proteins are key regulators of many cellular processes, suggesting an even broader impact on the expression.

The binding of MetRS within the coding region suggests that binding inhibits ribosome transit along the mRNA, thereby affecting the elongation rate. In such a case, one would expect to see an increase in ribosome density upstream to the MetRS binding site. However, a previous Ribosome Density Mapping along *YEF3* mRNA from cells grown under similar conditions did not reveal a higher density of ribosomes at that region compared to other regions along the mRNA (55). This suggests that impact is not through an elongation arrest. Due to the lack of apparent impact on elongation, we speculate that under normal conditions, the pseudouridine at position 1074 affects the translation initiation rate. How a modification that is located in the midst of an ORF affects events at the 5′ end of the transcript is yet to be revealed.

Yef3 protein is an elongation factor that assists the ribosomes’ movement along the ORF and plays a role in ribosome recycling (56). It may also play a non-translational role (57). Recently, the impact of Yef3p depletion on ribosome density was studied at high resolution (58). Interestingly, the depletion of Yef3p led to a higher representation of methionine codons at the P-site of stalled ribosomes. This implies a preferred role for Yef3p in assisting ribosomes stalled on these codons. Reduction in charged tRNA^Met^ levels may lead to this situation. Therefore, we speculate that under some conditions, the activity of Pus6 is repressed, leading to lower charging of tRNA^Met^ and ribosomes’ stalling. This condition will entail an increased need for Yef3p to rescue stalled ribosomes. This reiterates the tight interconnections between gene-specific and global regulation mediated by Pus6 and MetRS.

## Supplementary Material

gkaa1178_Supplemental_Files

## References

[1] Boccaletto P., Machnicka M.A., Purta E., Piatkowski P., Baginski B., Wirecki T.K., de Crécy-Lagard V., Ross R., Limbach P.A., Kotter A. et al. MODOMICS: a database of RNA modification pathways. 2017 update. Nucleic Acids Res. 2018; 46:D303–D307. 29106616 10.1093/nar/gkx1030 PMC5753262

[2] Davis F.F., Allen F.W. Ribonucleic acids from yeast which contain a fifth nucleotide. J. Biol. Chem. 1957; 227:907–915. 13463012

[3] Cohn W.E. 5-Ribosyl uracil, a carbon-carbon ribofuranosyl nucleoside in ribonucleic acids. BBA - Biochim. Biophys. Acta. 1959; 32:569–571. 13811055 10.1016/0006-3002(59)90644-4

[4] Durant P.C., Davis D.R. The effect of pseudouridine and pH on the structure and dynamics of the anticodon stem-loop of tRNA(Lys,3). Nucleic Acids Symp. Ser. 1997; 56–57. 9478205

[5] Kierzek E., Malgowska M., Lisowiec J., Turner D.H., Gdaniec Z., Kierzek R. The contribution of pseudouridine to stabilities and structure of RNAs. Nucleic. Acids. Res. 2014; 42:3492–3501. 24369424 10.1093/nar/gkt1330 PMC3950712

[6] Ofengand J. Ribosomal RNA pseudouridines and pseudouridine synthases. FEBS Letters. 2002; 514:John Wiley & Sons, Ltd 17–25. 11904174 10.1016/s0014-5793(02)02305-0

[7] Gray M., Charette M.W. Pseudouridine in RNA: what, where, how, and why. IUBMB Life (Int. Union Biochem. Mol. Biol. Life). 2000; 49:341–351. 10.1080/152165400410182 10902565

[8] Watkins N.J., Gottschalk A., Neubauer G., Kastner B., Fabrizio P., Mann M., Lührmann R. Cbf5p, a potential pseudouridine synthase, and Nhp2p, a putative RNA- binding protein, are present together with Gar1p in all H BOX/ACA-motif snoRNPs and constitute a common bipartite structure. RNA. 1998; 4:1549–1568. 9848653 10.1017/s1355838298980761 PMC1369725

[9] Lafontaine D.L., Bousquet-Antonelli C., Henry Y., Caizergues-Ferrer M., Tollervey D. The box H + ACA snoRNAs carry Cbf5p, the putative rRNA pseudouridine synthase. Genes Dev. 1998; 12:527–537. 9472021 10.1101/gad.12.4.527 PMC316522

[10] Rintala-Dempsey A.C., Kothe U. Eukaryotic stand-alone pseudouridine synthases–RNA modifying enzymes and emerging regulators of gene expression. RNA Biol. 2017; 14:1185–1196. 28045575 10.1080/15476286.2016.1276150 PMC5699540

[11] Behm-Ansmant I., Grosjean H., Massenet S., Motorin Y., Branlant C. Pseudouridylation at position 32 of mitochondrial and cytoplasmic tRNAs requires two distinct enzymes in Saccharomyces cerevisiae. J. Biol. Chem. 2004; 279:52998–53006. 15466869 10.1074/jbc.M409581200

[12] Behm-Ansmant I., Urban A., Ma X., Yu Y.T., Motorin Y., Branlant C. The Saccharomyces cerevisiae U2 snRNA:pseudouridine-synthase Pus7p is a novel multisite-multisubstrate RNA:ψ-synthase also acting on tRNAs. RNA. 2003; 9:1371–1382. 14561887 10.1261/rna.5520403 PMC1287059

[13] Lecointe F., Simos G., Sauer A., Hurt E.C., Motorin Y., Grosjean H. Characterization of yeast protein Deg1 as pseudouridine synthase (Pus3) catalyzing the formation of Ψ38 and Ψ39 in tRNA anticodon loop. J. Biol. Chem. 1998; 273:1316–1323. 9430663 10.1074/jbc.273.3.1316

[14] Ansman I., Motorin Y., Massenet S., Grosjean H., Branlant C. Identification and characterization of the tRNA:Ψ 31-synthase (Pus6p) of Saccharomyces cerevisiae. J. Biol. Chem. 2001; 276:34934–34940. 11406626 10.1074/jbc.M103131200

[15] Becker H., Motorin Y., Planta R.J., Grosjean H. The yeast gene YNL292w encodes a pseudouridine synthase (Pus4) catalyzing the formation of psi55 in both mitochondrial and cytoplasmic tRNAs. Nucleic Acids Res. 1997; 25:4493–4499. 9358157 10.1093/nar/25.22.4493 PMC147073

[16] Großhans H., Lecointe F., Grosjean H., Hurt E., Simos G. Pus1p-dependent tRNA pseudouridinylation becomes essential when tRNA Biogenesis is compromised in yeast. J. Biol. Chem. 2001; 276:46333–46339. 11571299 10.1074/jbc.M107141200

[17] Carlile T.M., Rojas-Duran M.F., Zinshteyn B., Shin H., Bartoli K.M., Gilbert W.V. Pseudouridine profiling reveals regulated mRNA pseudouridylation in yeast and human cells. Nature. 2014; 515:143–146. 25192136 10.1038/nature13802 PMC4224642

[18] Lovejoy A.F., Riordan D.P., Brown P.O. Transcriptome-wide mapping of pseudouridines: pseudouridine synthases modify specific mRNAs in S. cerevisiae. PLoS One. 2014; 9:e110799. 25353621 10.1371/journal.pone.0110799 PMC4212993

[19] Schwartz S., Bernstein D.A., Mumbach M.R., Jovanovic M., Herbst R.H., León-Ricardo B.X., Engreitz J.M., Guttman M., Satija R., Lander E.S. et al. Transcriptome-wide mapping reveals widespread dynamic-regulated pseudouridylation of ncRNA and mRNA. Cell. 2014; 159:148–162. 25219674 10.1016/j.cell.2014.08.028 PMC4180118

[20] Eyler D.E., Franco M.K., Batool Z., Wu M.Z., Dubuke M.L., Dobosz-Bartoszek M., Jones J.D., Polikanov Y.S., Roy B., Koutmou K.S. Pseudouridinylation of mRNA coding sequences alters translation. Proc. Natl. Acad. Sci. U.S.A. 2019; 116:23068–23074. 31672910 10.1073/pnas.1821754116 PMC6859337

[21] Wu G., Adachi H., Ge J., Stephenson D., Query C.C., Yu Y. Pseudouridines in U2 snRNA stimulate the ATPase activity of Prp5 during spliceosome assembly. EMBO J. 2016; 35:654–667. 26873591 10.15252/embj.201593113 PMC4801943

[22] Pang Y.L.J., Poruri K., Martinis S.A. tRNA synthetase: TRNA aminoacylation and beyond. Wiley Interdiscip. Rev. RNA. 2014; 5:461–480. 24706556 10.1002/wrna.1224 PMC4062602

[23] Guo M., Yang X.L., Schimmel P. New functions of aminoacyl-tRNA synthetases beyond translation. Nat. Rev. Mol. Cell Biol. 2010; 11:668–674. 20700144 10.1038/nrm2956 PMC3042954

[24] Levi O., Garin S., Arava Y. RNA mimicry in post-transcriptional regulation by aminoacyl tRNA synthetases. Wiley Interdiscip. Rev. RNA. 2019; 11:e1564. 31414576 10.1002/wrna.1564

[25] Melnikov S.V., Söll D. Aminoacyl-tRNA synthetases and tRNAS for an expanded genetic code: what makes them orthogonal. Int. J. Mol. Sci. 2019; 20:1929. 10.3390/ijms20081929 PMC6515474 31010123

[26] Lorenz C., Lünse C.E., Mörl M. tRNA modifications: impact on structure and thermal adaptation. Biomolecules. 2017; 7:35. 10.3390/biom7020035 PMC5485724 28375166

[27] Liu S., Comandur R., Jones C.P., Tsang P., Musier-Forsyth K. Anticodon-like binding of the HIV-1 tRNA-like element to human lysyl-tRNA synthetase. RNA. 2016; 22:1828–1835. 27852925 10.1261/rna.058081.116 PMC5113203

[28] Kämper U., Kück U., Cherniack A.D., Lambowitz A.M. The mitochondrial tyrosyl-tRNA synthetase of Podospora anserina is a bifunctional enzyme active in protein synthesis and RNA splicing. Mol. Cell. Biol. 1992; 12:499–511. 1531084 10.1128/mcb.12.2.499-511.1992 PMC364206

[29] Beckmann B.M., Horos R., Fischer B., Castello A., Eichelbaum K., Alleaume A.M., Schwarzl T., Curk T., Foehr S., Huber W. et al. The RNA-binding proteomes from yeast to man harbour conserved enigmRBPs. Nat. Commun. 2015; 6:10127. 26632259 10.1038/ncomms10127 PMC4686815

[30] Matia-González A.M., Laing E.E., Gerber A.P. Conserved mRNA-binding proteomes in eukaryotic organisms. Nat. Struct. Mol. Biol. 2015; 22:1027–1033. 26595419 10.1038/nsmb.3128 PMC5759928

[31] Mitchell S.F., Jain S., She M., Parker R. Global analysis of yeast mRNPs. Nat. Struct. Mol. Biol. 2013; 20:127–133. 23222640 10.1038/nsmb.2468 PMC3537908

[32] Romby P., Brunel C., Caillet J., Springer M., Grunberg-Manago M., Westhof E., Ehresmann C., Ehresmann B. Molecular mimicry in translational control of E. coli threonyl-tRNA synthetase gene. Competitive inhibition in tRNA aminoacylation and operator-repressor recognition switch using tRNA identity rules. Nucleic Acids Res. 1992; 20:5633–5640. 1280807 10.1093/nar/20.21.5633 PMC334396

[33] Levi O., Arava Y. mRNA association by aminoacyl tRNA synthetase occurs at a putative anticodon mimic and autoregulates translation in response to tRNA levels. PLoS Biol. 2019; 17:e3000274. 31100060 10.1371/journal.pbio.3000274 PMC6542539

[34] Jeong S.J., Park S., Nguyen L.T., Hwang J., Lee E.Y., Giong H.K., Lee J.S., Yoon I., Lee J.H., Kim J.H. et al. A threonyl-tRNA synthetase-mediated translation initiation machinery. Nat. Commun. 2019; 10:1357. 30902983 10.1038/s41467-019-09086-0 PMC6430810

[35] Torrent M., Chalancon G., De Groot N.S., Wuster A., Madan Babu M. Cells alter their tRNA abundance to selectively regulate protein synthesis during stress conditions. Sci. Signal. 2018; 11:eaat6409. 30181241 10.1126/scisignal.aat6409 PMC6130803

[36] Huh W.-K., Falvo J.V., Gerke L.C., Carroll A.S., Howson R.W., Weissman J.S., O’Shea E.K. Global analysis of protein localization in budding yeast. Nature. 2003; 425:686–691. 14562095 10.1038/nature02026

[37] Weill U., Yofe I., Sass E., Stynen B., Davidi D., Natarajan J., Ben-Menachem R., Avihou Z., Goldman O., Harpaz N. et al. Genome-wide SWAp-Tag yeast libraries for proteome exploration. Nat. Methods. 2018; 15:617–622. 29988094 10.1038/s41592-018-0044-9 PMC6076999

[38] Baker Brachmann C., Davies A., Cost G.J., Caputo E., Li J., Hieter P., Boeke J.D. Designer deletion strains derived from Saccharomyces cerevisiae S288C: a useful set of strains and plasmids for PCR-mediated gene disruption and other applications. Yeast. 1998; 14:115–132. 9483801 10.1002/(SICI)1097-0061(19980130)14:2<115::AID-YEA204>3.0.CO;2-2

[39] Haramati O., Brodov A., Yelin I., Atir-Lande A., Samra N., Arava Y. Identification and characterization of roles for Puf1 and Puf2 proteins in the yeast response to high calcium. Sci. Rep. 2017; 7:3037. 28596535 10.1038/s41598-017-02873-z PMC5465220

[40] Zhang Y., Liu T., Meyer C.A., Eeckhoute J., Johnson D.S., Bernstein B.E., Nussbaum C., Myers R.M., Brown M., Li W. et al. Model-based analysis of ChIP-Seq (MACS). Genome Biol. 2008; 9:R137. 18798982 10.1186/gb-2008-9-9-r137 PMC2592715

[41] Dekker B., Anand R., Memisoglu G., Haber J. Cas9-mediated gene editing in Saccharomyces cerevisiae. Protoc. Exch. 2017; doi:10.1038/protex.2017.021a.

[42] Levi O., Arava Y. Expanding the CRISPR/Cas9 toolbox for gene engineering in S. cerevisiae. Curr. Microbiol. 2020; 77:468–478. 31901956 10.1007/s00284-019-01851-0

[43] Eldad N., Yosefzon Y., Arava Y. Identification and characterization of extensive intra-molecular associations between 3′-UTRs and their ORFs. Nucleic. Acids. Res. 2008; 36:6728–6738. 18948291 10.1093/nar/gkn754 PMC2588509

[44] Bailey T.L., Johnson J., Grant C.E., Noble W.S. The MEME suite. Nucleic. Acids. Res. 2015; 43:W39–W49. 25953851 10.1093/nar/gkv416 PMC4489269

[45] Chan P.P., Lowe T.M. GtRNAdb 2.0: an expanded database of transfer RNA genes identified in complete and draft genomes. Nucleic Acids Res. 2016; 44:D184–D189. 26673694 10.1093/nar/gkv1309 PMC4702915

[46] Helm M., Motorin Y. Detecting RNA modifications in the epitranscriptome: predict and validate. Nat. Rev. Genet. 2017; 18:275–291. 28216634 10.1038/nrg.2016.169

[47] Zaringhalam M., Papavasiliou F.N. Pseudouridylation meets next-generation sequencing. Methods. 2016; 107:63–72. 26968262 10.1016/j.ymeth.2016.03.001

[48] Nakamura Y., Gojobori T., Ikemura T. Codon usage tabulated from international DNA sequence databases: status for the year 2000. Nucleic Acids Res. 2000; 28:292. 10592250 10.1093/nar/28.1.292 PMC102460

[49] Liu Y. A code within the genetic code: codon usage regulates co-translational protein folding. Cell Commun. Signal. 2020; 18:145. 32907610 10.1186/s12964-020-00642-6 PMC7488015

[50] Yakobov N., Debard S., Fischer F., Senger B., Becker H.D. Cytosolic aminoacyl-tRNA synthetases: unanticipated relocations for unexpected functions. Biochim. Biophys. Acta - Gene Regul. Mech. 2018; 1861:387–400. 29155070 10.1016/j.bbagrm.2017.11.004

[51] Sampath P., Mazumder B., Seshadri V., Gerber C.A., Chavatte L., Kinter M., Ting S.M., Dignam J.D., Kim S., Driscoll D.M. et al. Noncanonical function of glutamyl-prolyl-tRNA synthetase. Cell. 2004; 119:195–208. 15479637 10.1016/j.cell.2004.09.030

[52] Hinnebusch A.G. Translational regulation of GCN4 and the general amino acid control of yeast. Annu. Rev. Microbiol. 2005; 59:407–450. 16153175 10.1146/annurev.micro.59.031805.133833

[53] Wek R.C., Jackson B.M., Hinnebusch A.G. Juxtaposition of domains homologous to protein kinase and histidyl-tRNA synthetases in GCN2 protein suggests a mechanism for coupling GCN4 expression to amino acid availability. Proc. Natl. Acad. Sci. U.S.A. 1989; 86:4579–4583. 2660141 10.1073/pnas.86.12.4579 PMC287314

[54] Vazquez de Aldana C.R., Wek R.C., Segundo P.S., Truesdell A.G., Hinnebusch A.G. Multicopy tRNA genes functionally suppress mutations in yeast eIF-2 alpha kinase GCN2: evidence for separate pathways coupling GCN4 expression to unchanged tRNA. Mol. Cell. Biol. 1994; 14:7920–7932. 7969132 10.1128/mcb.14.12.7920-7932.1994 PMC359331

[55] Arava Y., Boas F.E., Brown P.O., Herschlag D. Dissecting eukaryotic translation and its control by ribosome density mapping. Nucleic. Acids. Res. 2005; 33:2421–2432. 15860778 10.1093/nar/gki331 PMC1087779

[56] Dever T.E., Green R. The elongation, termination, and recycling phases of translation in eukaryotes. Cold Spring Harb. Perspect. Biol. 2012; 4:a013706. 22751155 10.1101/cshperspect.a013706 PMC3385960

[57] Samra N., Atir-Lande A., Pnueli L., Arava Y. The elongation factor eEF3 (Yef3) interacts with mRNA in a translation independent manner. BMC Mol. Biol. 2015; 16:17. 26404137 10.1186/s12867-015-0045-5 PMC4582935

[58] Kasari V., Margus T., Atkinson G.C., Johansson M.J.O., Hauryliuk V. Ribosome profiling analysis of eEF3-depleted Saccharomyces cerevisiae. Sci. Rep. 2019; 9:3037. 30816176 10.1038/s41598-019-39403-y PMC6395859

[59] Bailey T.L. DREME: motif discovery in transcription factor ChIP-seq data. Bioinformatics. 2011; 27:1653–1659. 21543442 10.1093/bioinformatics/btr261 PMC3106199

