# Supplementary material for: Pseudouridine-mediated translation control of mRNA by methionine aminoacyl tRNA synthetase
Source: Nucleic Acids Res. 2020 Dec 10;49(1):432–43. doi: 10.1093/nar/gkaa1178 (PMC7797078; doi:10.1093/nar/gkaa1178)
Supplement: gkaa1178_Supplemental_Files [file gkaa1178_supplemental_files.zip › Supplementary Figures and Tables_corrected.pdf]

Supplementary Figures and Tables

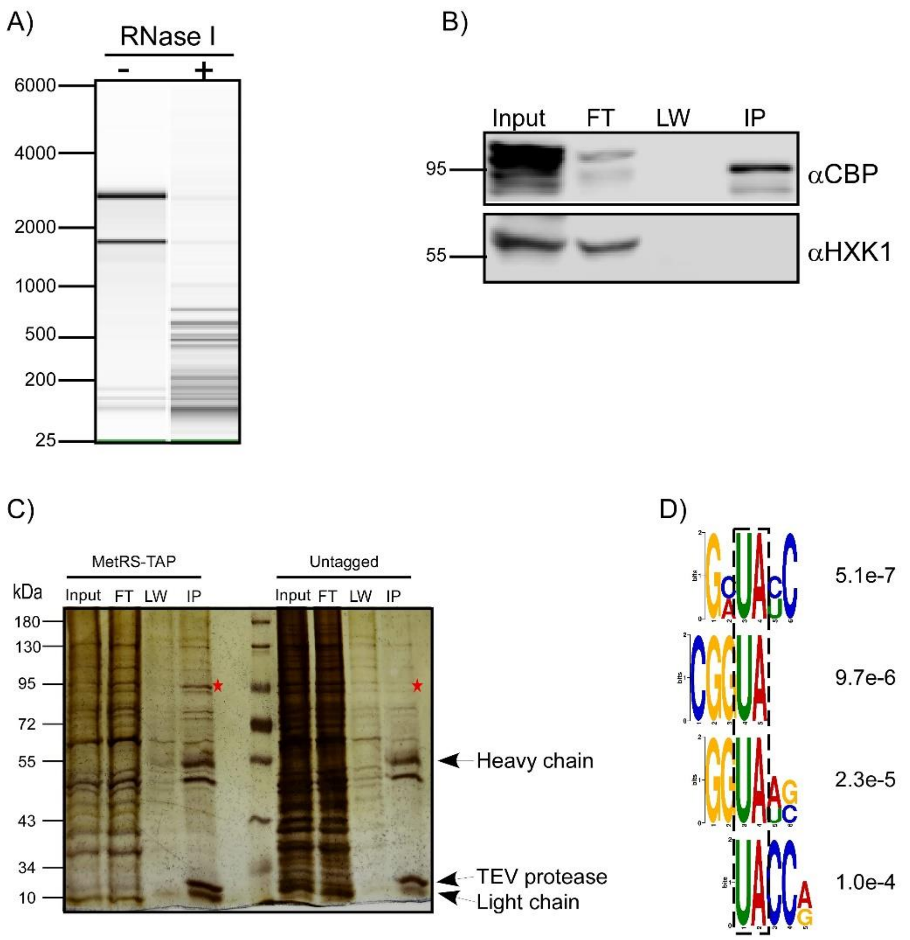

Supplementary Figure 1: fragmentation RIP-Seq controls. A) Efficiency of fragmentation. RNA samples from MetRS-TAP either subjected to fragmentation by RNase I (+) or not (-) were resolved on Agilent 2100 Bioanalyzer using Eukaryotic Total RNA Pico kit. B) Specificity of isolation. TAP-tagged MetRS strain was subjected to RIP using IgG Sepharose beads. Protein samples from the total lysate (2.5% of sample) (Input), unbound sample (2.5%)(FT), last wash (2.5%)(LW), and the TEV-eluted fraction (2.5%)(IP) were resolved on SDS-PAGE followed by western analysis with the indicated antibodies. Note that following cleavage of the proteinA domain, the eluted protein (recognized by the antibody to calmodulin binding protein domain (αCBP)) is shortened. C) Purity of isolation. Protein samples from the TAP-tagged strain and the untagged parental strain were collected from the cellular lysate (0.3%)(Input), flow through (0.3%)(FT) step, last wash of the beads (10%)(LW) and bound material (10%)(IP). Samples were resolved on PAGE and stained by Silver staining. The expected position of MetRS-TAP is indicated by an asterisk. Also indicated IgG light and heavy chains that are released from the beads and the TEV protease that is added for elution. D) Logo presentation of top motifs identified by DREME within the fragments associated by MetRS. P values are indicated to the right of each motif.

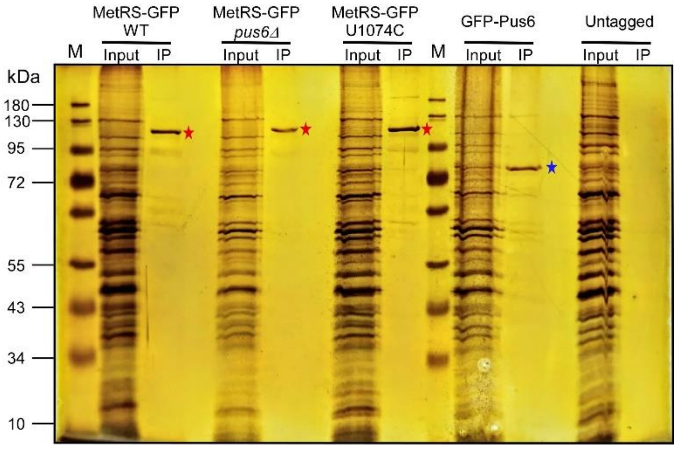

Supplementary Figure 2: Similar purification efficiency for various tagged proteins. The indicated GFP-tagged proteins were subjected to RIP protocol using GFPtrap beads (Chromotek). Protein sample from the cellular lysate (Input) and from the material eluted from the beads (IP) were analyzed by Silver staining. The tagged proteins are indicated by an asterisk.

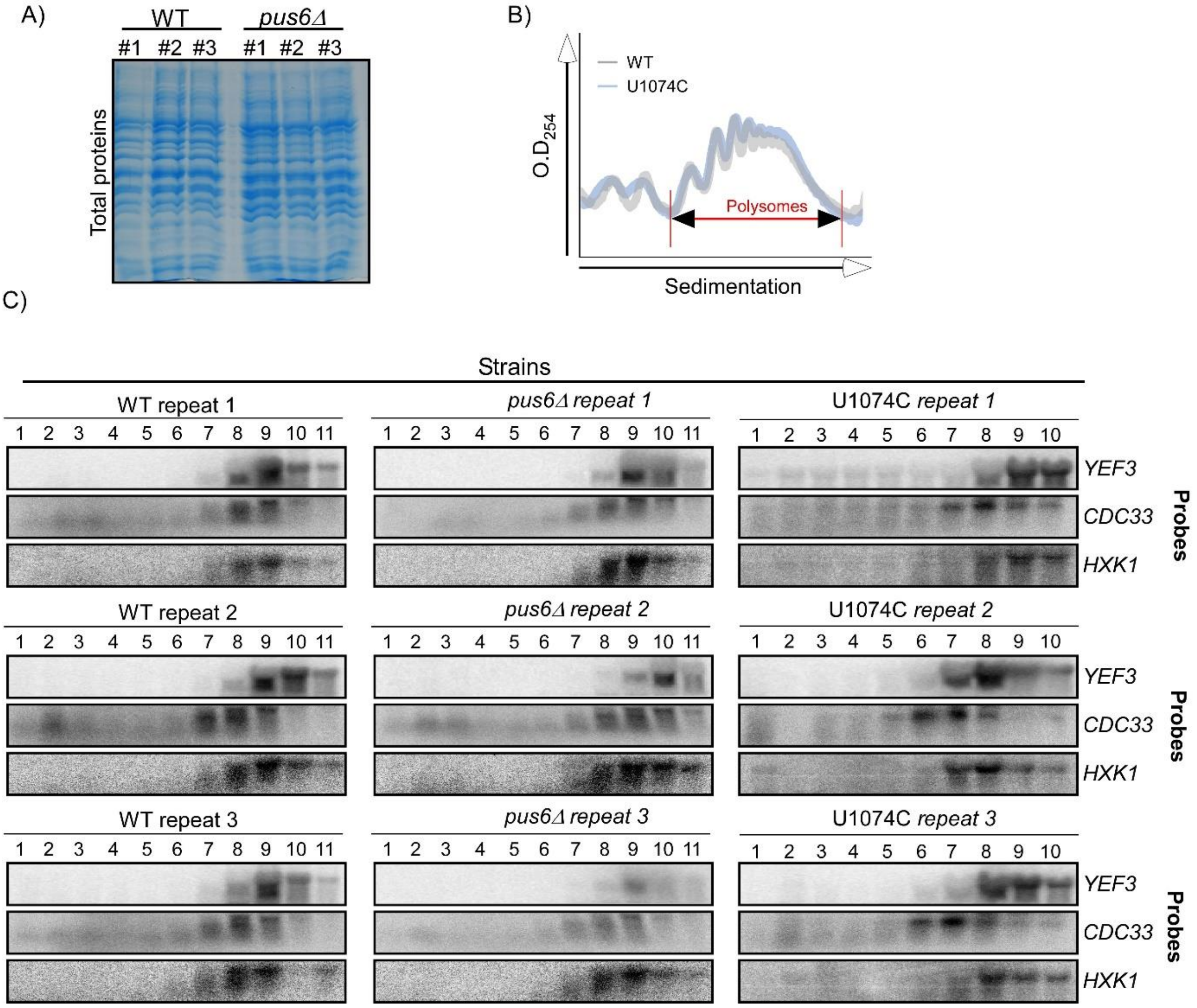

Supplementary Figure 3: Protein and polysomal profiles. A) Commassie blue staining of protein samples, extracted from three independent cultures of WT and *pusΔ* cells grown to the same OD600. B) WT and U1074C cells were subjected to polysomal analysis on sucrose gradients. OD254 was monitored throughout the gradient, and the sedimentation position of polysomal complexes (>2 ribosomes) is indicated. C) Fractions were collected from the entire gradients, RNA was purified from each fraction and subjected to northern analysis with the probes indicated to the right. Blots from three independent biological repeats, for WT, *pus6Δ* and U1074C strains are presented. Quantification results are presented in Figures 4C and 7F.

Supplementary Tables

Supplementary Table 1
List of primers used in this study.

**Oligos for PCR amplification of Northern probe**	
YEF3 F	GGTCGAAGACCCACAAGTTATT
YEF3 R	CCACCAGACAAAGCAGAGATT
CDC33 F	AAAGCCAGCCGTCGATAAA
CDC33 R	ATTGATGGTTGAGGGTGTCTAC
HXK1 F	AATGTCGAAGGCCACGATGT
HXK1 R	CTTTGCTTGCGTCACCAGTC
**gRNA Oligos**	
**Name**	**Sequence**
YEF3 1063 gRNA F	AATCTTAAATCTTGGAGCAAGTTTT
YEF3 1063 gRNA R	TTGCTCCAAGATTTAAGATTGATCA
**Donor DNA Oligos**	
**Name**	**Sequence**
Yef3 T1074C F	TAACGAATTGTTGAAGGACGAAACCGTTGCTCCAAGATTCAAGAT TGTCGTCGAGTACATTGCCGCCATTGGTGCTGAT
Yef3 T1074C R	ATCAGCACCAATGGCGGCAATGTACTCGACGACAATCTTGAATCT TGGAGCAACGGTTTCGTCCTTCAACAATTCGTTAA
**qPCR Oligos**	
**Name**	**Sequence**
YEF3 qPCR F (+2304)	CATGGACAGAGCTAACAGACAA
YEF3 qPCR R (+2404)	GTGGATACCGGCAATTCTTCTA
CDC33 qPCR F (+46)	GTCGATGATACCACAGCTACTC
CDC33 R qPCR (+182)	GATCAGACCACGACTCAGATTTA
ACT1 qPCR F (+4)	GATTCTGAGGTTGCTGCTTTG
ACT1 qPCR R (+109)	ACCGACGATAGATGGGAAGA
tRNA-Pro_AGG F	GGCGTGTGGTCTAGAGGTATG
tRNA-Pro_AGG R	ACTCGAACCCGGGACCTC
tRNA-eMet F	GCTCAGTAGGAAGAGCGTCA
tRNA-eMet R	GGTTCGAACTCTCGACCTTCA

Supplementary Table 2 (in a separate excel file)
MetRS bound mRNA regions. Excel file showing MACS2 callpeak results including: location, Fold Change and statistical significance of each peak. This file includes two sheets. **Sheet 1**: All peaks detected by MACS call peak, for MetRS fRIP-seq experiments assigned within transcribed mRNAs. **Sheet 2**: regions significantly bound by MetRS, using selection criteria as described in Materials and Methods.

Supplementary Table 3 (in a separate excel file)
GO Term analyses results for genes of interest (using SGD GO Term Finder Version 0.86).

Supplementary Table 4 (in a separate excel file)
List of MetRS bound regions that contain pseudouridine site.
